# Colonization and Infection of the Skin by *S. aureus*: Immune System Evasion and the Response to Cationic Antimicrobial Peptides

**DOI:** 10.3390/ijms15058753

**Published:** 2014-05-16

**Authors:** Sunhyo Ryu, Peter I. Song, Chang Ho Seo, Hyeonsook Cheong, Yoonkyung Park

**Affiliations:** 1 Department of Biotechnology, Chosun University, Gwangju 501-759, Korea; E-Mails: bktsh0328@gmail.com (S.R.); hscheong@chosun.ac.kr (H.C.); 2 Department of Dermatology, University of Arkansas for Medical Sciences, Little Rock, AR 72205, USA; E-Mail: psong@uams.edu; 3 Department of Bioinformatics, Kongju National University, Kongju 314-701, Korea; E-Mail: chseo@kongju.ac.kr

**Keywords:** *Staphylococcus aureus*, skin colonization, skin infection, immune system evasion, cationic antimicrobial peptides (CAMPs)

## Abstract

*Staphylococcus aureus* (*S. aureus*) is a widespread cutaneous pathogen responsible for the great majority of bacterial skin infections in humans. The incidence of skin infections by *S. aureus* reflects in part the competition between host cutaneous immune defenses and *S. aureus* virulence factors. As part of the innate immune system in the skin, cationic antimicrobial peptides (CAMPs) such as the β-defensins and cathelicidin contribute to host cutaneous defense, which prevents harmful microorganisms, like *S. aureus*, from crossing epithelial barriers. Conversely, *S. aureus* utilizes evasive mechanisms against host defenses to promote its colonization and infection of the skin. In this review, we focus on host-pathogen interactions during colonization and infection of the skin by *S. aureus* and methicillin-resistant *Staphylococcus aureus* (MRSA). We will discuss the peptides (defensins, cathelicidins, RNase7, dermcidin) and other mediators (toll-like receptor, IL-1 and IL-17) that comprise the host defense against *S. aureus* skin infection, as well as the various mechanisms by which *S. aureus* evades host defenses. It is anticipated that greater understanding of these mechanisms will enable development of more sustainable antimicrobial compounds and new therapeutic approaches to the treatment of *S. aureus* skin infection and colonization.

## 1. Introduction

*Staphylococcus aureus* (*S. aureus*) is a Gram-positive bacterium that can live as a commensal organism on the skin and in the nose and throat. Approximately 30% of healthy people are asymptomatically colonized by *S. aureus*, which permanently colonizes the anterior nares in 10%–20% of the population and intermittently colonizes 30%–50%; the rest of the population never becomes colonized [1,2]. Importantly, this colonization is a known risk factor for infection [3–7], and *S. aureus* causes a range of infections, from minor skin infections to abscesses, endocarditis and sepsis. *S. aureus* is also a major cause of food poising induced by heat resistant enterotoxin A and is a leading cause of nosocomial infections [2], as colonized healthcare workers can transmit the pathogen to immunosuppressed patients. In addition, several cases of community-acquired methicillin-resistant *S. aureus* (CA-MRSA) infections have been recently reported [8–10]. Notably, these reports describe severe and even lethal infections by highly virulent strains of *S. aureus* in immunocompetent individuals.

*S. aureus* is exposed to a large arsenal of highly efficient antimicrobial host factors during skin colonization and infection. However, a growing number of dedicated resistance mechanisms now contribute to the ability of *S. aureus* to evade host cutaneous defenses and survive during colonization [11,12]. Furthermore, Glaser *et al.* recently reported that *S. aureus* small colony variants (SCVs) are less susceptible to the bactericidal activity of different human skin-derived AMP, which are associated with a higher resistance to the killing activity of human stratum corneum [13]. Both host cutaneous defense mechanisms and *S. aureus* virulence factors appear to be the focus of actively ongoing co-evolution, leading to major variations between different host species and bacterial strains, respectively [14,15]. Understanding how ones immune system combats the evasion strategies of *S. aureus* could be useful for the development of novel and more sustainable antimicrobial agents that are not subject to the evolution of microbial resistance.

While bacterial resistance to most available antibiotics is increasing and our knowledge about the arsenal of host cutaneous defense strategies is growing, it is becoming increasingly attractive to consider endogenous antimicrobial peptides (AMPs) as sources for more sustainable antimicrobial agents. Of the variety of host defense molecules expressed by organisms, cationic AMPs (CAMPs) have proven to be particularly promising for future development as new antimicrobials. This review focuses on the role of host CAMPs in staphylococcal skin infections, and on the mechanisms underlying *S. aureus* resistance to CAMPs.

## 2. Host-Pathogen Interactions during *S. aureus* Skin Colonization and Infection

The epidermis is composed of proliferating basal and differentiated suprabasal keratinocytes, within which sweat glands, sebaceous glands and hair follicles are sparsely distributed. Langerhans cells in the epidermis as well as dendritic cells, macrophages, mast cells, T and B cells, plasma cells and natural killer cells in the dermis participate in immune responses within the skin. As mentioned, approximately 30% of healthy individuals are colonized by *S. aureus* [16] through a process that reflects the competition between host factors and commensal organisms that resist colonization and *S. aureus* virulence factors that facilitate colonization and, possibly, subsequent infection [17]. Among the constitutive properties of skin that help to prevent colonization and infection by *S. aureus* are its low temperature and acidic pH [18,19]. For instance, an epidermal structural component, filaggrin, is broken down during epidermal differentiation into urocanic acid and pyrrolidone carboxylic acid [20]. These acidic breakdown products then not only contribute to the low pH of the skin surface but also inhibit the growth of *S. aureus* and the expression of at least two factors involved in *S. aureus* colonization, clumping factor B (ClfB) and fibronectin binding protein A (FnbpA) [20]. In addition, commensal organisms such as *S. epidermidis*, *P. acnes* and the *Malassezia* species are normally present on the skin surface occupying microbial niches and thus preventing colonization and invasion by *S. aureus* and other pathogens [18,19]. Skin commensals have also been shown to directly inhibit *S. aureus* colonization of skin and nasal mucosa. For example, *S. epidermidis* secretes a serine protease, Esp, which inhibits *S. aureus* colonization by destroying its biofilms [21]. *S. epidermidis* also produces phenol-soluble modulins (PSMγ and PSMδ), which have direct antimicrobial activity against *S. aureus* [22] and activate toll-like receptor 2 (TLR2) on keratinocytes, leading to production of CAMPs (e.g., human β-defensin 2 [hBD2], hBD3 and RNase 7), which amplify the immune response and promote killing of *S. aureus* [23,24]. CAMPs such as hBD2, hBD3, LL-37 (cathelicidin) and RNase 7, which are produced by keratinocytes in the skin and corneal layer, have bacteriostatic or bactericidal activity against *S. aureus* [25–28], as evidenced by the observation that *S. aureus* colonization is increased in skin lesions caused by atopic dermatitis due to reductions in the levels of β-defensins and cathelicidin [29].

To promote colonization of human nasal mucosa and skin, *S. aureus* expresses various factors that facilitate skin surface binding and survival. To bind to host surface components such as fibrinogen, fibronectin and cytokeratins, which are derived from epidermal keratinocytes or nasal epithelium, *S. aureus* utilizes microbial surface components recognizing adhesive matrix molecules (MSCRAMMs), which include fibronectin-binding protein A (Fnbp A) and Fnbp B, fibrinogen-binding proteins (ClfA and ClfB), iron-regulated surface determinant A (IsdA) and wall teichoic acid [30–33]. *S. aureus*-mediated fibronectin and fibrinogen binding is also enhanced by elevated levels of Th2 cytokines. For example, interleukin (IL)-4 is elevated in the skin lesions of atopic dermatitis patients, which are highly susceptible to *S. aureus* colonization [31]. *S. aureus* also produces superantigens such as staphylococcal enterotoxins A and B (SEA and SEB) and toxic shock syndrome toxin-1 (TSST-1), which skew the cutaneous immune response towards the Th2 cytokines and thus contribute to the increased colonization of *S. aureus* in atopic dermatitis patients [34]. In addition, *S. aureus* expresses factors that enable it to directly counter host CAMP responses. For example, IsdA enhances bacterial cellular hydrophobicity, which renders *S. aureus* resistant to bactericidal fatty acids in sebum and to β-defensins and cathelicidin [35]. It also secretes a protein, aureolysin, which is an extracellular metalloproteinase that inhibits cathelicidin antimicrobial activity [36]. Virulence factors from *S. aureus* are also closely related with evasion from human innate immune defenses [37]. In the following section, the mechanisms by which *S. aureus* inhibits the activities of CAMPs will be described in detail.

## 3. Methicillin-Resistant *S. aureus* (MRSA) Infection

Antibiotic resistance is now recognized to be a serious hindrance to the management of *S. aureus*. For instance, β-lactam antibiotics (e.g., methicillin) have proven unfavorable for the management of toxic *S. aureus* infections, because even subinhibitory concentrations lead to increased expression of α-toxin through a stimulatory effect on exoprotein synthesis [38–40]. Instead, protein-synthesis-suppressing antibiotics such as clindamycin and linezolid are recommended for the treatment of *S. aureus*-induced toxicity syndromes, as concentrations below the MIC impair expression of *S. aureus* virulence factors [41,42]. Clindamycin at a concentration of 1/8 MIC inhibits the expression of α- and δ-haemolysin as well as coagulase [43]. In addition, the expression of protein A is reduced when *S. aureus* is exposed to clindamycin at concentrations below the MIC, leading to increased bacterial susceptibility to phagocytosis and suggesting additional therapeutic efficacy [44]. However, clindamycin cannot be used to treat toxic MRSA infections because MRSA is largely resistant to clindamycin.

MRSA infections are caused by strains of *S. aureus* that have become resistant to the antibiotics commonly used to treat ordinary infections. Most MRSA infections occur in people who have been in hospitals or other health care settings, such as nursing homes and dialysis centers. When it occurs in these settings, it is known as health care-associated MRSA (HA-MRSA). HA-MRSA infections are typically associated with invasive procedures or devices, such as surgeries, intravenous tubing or artificial joints. However, another type of MRSA infection occurs in the wider community, among otherwise healthy individuals. This form, community-associated MRSA (CA-MRSA) is spread by skin-to-skin contact. It often begins as a painful skin boil and generally causes skin and soft tissue infections, but it is also capable of causing invasive disease such as endocarditis, necrotizing pneumonia and sepsis [45–50]. HA-MRSA, by contrast, is considered a nosocomial pathogen typically associated with invasive disease, such as bloodstream infections, pneumonia, surgical site infections and urinary tract infections [45,51–53]. It is now recognized that these two entities are genetically distinct. Isolates of HA-MRSA are likely to be resistant to three or more antibiotic classes, whereas CA-MRSA is usually resistant only to β-lactams and macrolides [47,51,53,54].

Resistance to methicillin is mediated in *S. aureus* by PBP2a, a penicillin-binding protein with a low affinity for β-lactams. PBPs are membrane-bound enzymes that catalyze the transpeptidation reaction, which is necessary for cross-linkage of peptidoglycan chains [55]. PBP2a substitutes for the other PBPs and, because of its low affinity for all β-lactam antibiotics, enables staphylococci to survive exposure to high concentrations of these agents. Thus, resistance to methicillin confers resistance to all β-lactam agents, including cephalosporins. Expression of resistance in some MRSA strains is regulated by homologues of the regulatory genes for *blaZ*. These genes, *mecI* and *mecR1*, regulate the *mecA* response to β-lactam antibiotics in a fashion similar to the regulation of the *blaZ* response to penicillin by *blaR1* and *blaI*. Katayama *et al.* demonstrated that *mecA* is carried on a mobile genetic element and is part of a genomic island designated staphylococcal cassette chromosome *mec* (SCC*mec*) [56]. To date, four different SCC*mec* elements varying in size from 21 to 67 kb have been characterized [57]. Such islands may also contain additional genes for antimicrobial resistance and insertion sequences, as well as genes whose function is uncertain.

As *S. aureus* isolates from intensive care units and blood cultures have become increasingly resistant to greater numbers of antimicrobial agents [2,58], this has inevitably diminished the number of effective bactericidal antibiotics available to treat these often life-threatening infections. As rapidly as new antibiotics are introduced, staphylococci are developing efficient mechanisms to neutralize them. Recent reports of *S. aureus* isolates with intermediate or complete resistance to vancomycin portend a chemotherapeutic era in which effective bactericidal antibiotics against this organism may no longer be readily available [59,60]. Consequently the need to identify new alternative therapeutic targets and to develop novel drugs that can be used against these targets is increasing.

## 4. Human AMPs Effective against *S. aureus*

AMPs are a diverse group of polypeptides that are typically less than 50 amino acids in length and exhibit bactericidal activity under physiologic conditions [61–63]. Most AMPs are cationic and interact with the anionic microbial membrane leading to osmotic lysis [61–63]. Autolytic enzymes induced by AMPs may also be associated with bacterial cell death [64]. Whereas some AMPs are produced by keratinocytes and are normally present in the skin, others are induced during infection and inflammation/wounding [65,66]. In this section, we will focus on the specific bacteriostatic or bactericidal AMPs expressed by keratinocytes and by immune cells thought to contribute to host defense against *S. aureus* (Table 1).

These include α-defensins (also called human neutrophil peptides [HNPs]), β-defensins (hBD1-4) cathelicidin (LL-37), RNase7 and dermcidin [61–63]. These AMPs not only have bactericidal activity against *S. aureus*, they also promote the recruitment of immune cells to sites of infection. For example, HNPs promote recruitment of macrophages, T cells and mast cells through a PKC-dependent mechanism [85], while hBD2 and hBD3 promote CCR6-mediated chemotaxis of immature dendritic cells and memory CD4+ T cells and CCR2-mediated chemotaxis of monocytes/macrophages [86,87]. In addition, LL-37 promotes chemotaxis of neutrophils, monocytes and T cells by activating formyl peptide receptor-like 1 [88,89]. Through these various mechanisms, AMPs enhance host defenses against *S. aureus*.

### 4.1. Defensins

Neutrophils express high levels of HNP1-3 and lower levels of HNP4, which together constitute nearly 50% of the peptides within neutrophil granules [67]. HNP2 has the highest degree of bactericidal activity against *S. aureus*, though HNP1, 3 and 4 also exhibit some activity against *S. aureus* [68].

Most AMPs expressed in humans belong to the β-defensin family. These amphipathic peptides have a β-sheet structure and are subcategorized according to the number and location of their disulfide bridges [90]. Four well-characterized human β-defensins (hBD1-4) are expressed by epithelial cells, including keratinocytes, as well as by activated monocytes/macrophages and dendritic cells [62,63]. *hBD1* is constitutively expressed, while *hBD2* and *hBD3* are inducible by bacterial infection or cytokines [91]. hBD1 has no antimicrobial activity against *S. aureus*, while hBD2 and hBD4 show weak bacteriostatic activity against *S. aureus in vitro* [26,81]. By contrast, hBD3 exhibits strong bactericidal activity against *S. aureus in vitro* and in skin explants *ex vivo* [27,77]. In keratinocytes, production of hBD2, hBD3 and LL-37 can be induced by live or heat-killed *S. aureus* or by bacterial components such as lipopeptides and lipoteichoic acid [74,75,78–80]. Activation of the epidermal growth factor receptor through wounding of human skin also leads to increased hBD3 production, providing another mechanism for enhancing antimicrobial activity against *S. aureus* [66,92]. Finally, defensins induce production of cytokines, including IL-8, and have chemotactic activity [93].

### 4.2. Cathelicidins

Cathelicidins are a family of AMPs whose named reflects their resemblance to the precursor forms of the protein cathelin [94]. The *N*-terminal cathelin domain keeps the AMP precursor inactive until proteolytic cleavage releases the active *C*-terminal peptide. Although they may vary in structure, most mature cathelicidins are α-helical, amphipathic and cationic. Cathelicidin is constitutively expressed in neutrophils and has potent bactericidal activity against *S. aureus*. It is also called LL-37, referring its first two amino acids and total length of 37 amino acids [25,62,63]. Two other forms, RK-31 and KS-30, may be produced through alternative cleavage, especially on the skin [95]. Both KS-30 and RK-31 show greater antimicrobial activity than LL-37 and also differ from LL-37 in their ability to elicit cytokine release. Like defensins, LL-37 can induce both chemotaxis and cytokine release [93].

Vitamin D may also play a role in host defense against *S. aureus* skin infections, since it induces production of LL-37 in keratinocytes, neutrophils and monocytes/macrophages. However, the link between vitamin D and host defense against *S. aureus* has yet to be demonstrated in the skin [96–98].

### 4.3. RNase7

The cationic peptide RNase 7 is produced by many cell types, including keratinocytes, and has bactericidal activity against a broad range of bacteria, including *S. aureus* [61,99]. The high levels of RNase 7 present in the stratum corneum prevents *S. aureus* colonization of skin explants [28].

### 4.4. Dermcidin

Dermcidin is produced by human eccrine sweat glands and its processed forms have activity against numerous bacteria, including *S. aureus* [82,83,100,101]. While the DCD-1L and DCD-1 processed forms of dermcidin are negatively charged [69], a further processed cationic form (SSl-25) also shows antimicrobial activity, suggesting that charge is of no importance to the mode of action of dermcidin-derived peptides [83].

## 5. Cutaneous Host Defense Involving TLR-Mediated AMP Activity against *S. aureus*

**Figure 1.**
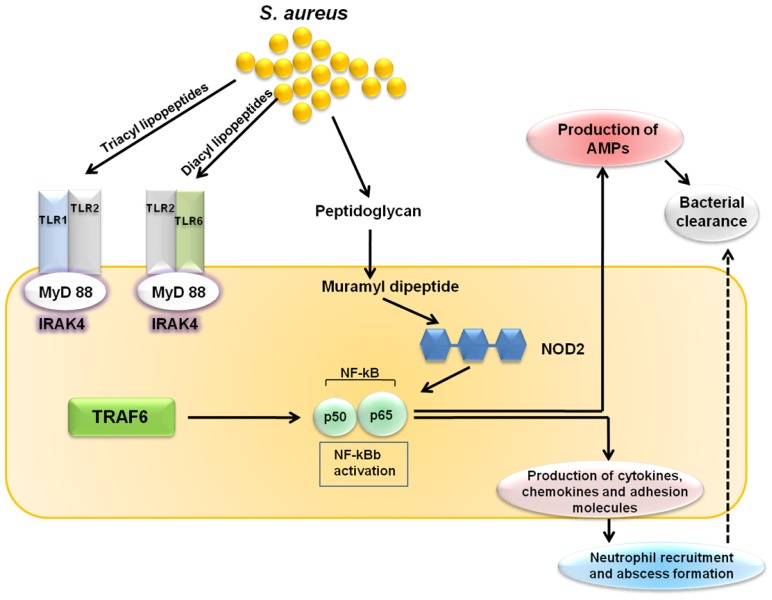
Toll-like receptor-mediated cutaneous immune response against *S. aureus*. Toll-like receptor 2 (TLR2) and nucleotide-binding oligomerization domain containing 2 (NOD2), which are expressed by keratinocytes, respectively recognize *S. aureus* lipopeptides/lipoteichoic acid and muramyl dipeptide. Both TLR2 and NOD2 signaling triggers the activation of nuclear factor-κB (NF-κB), which leads to the production of AMPs, cytokines, chemokines, adhesion molecules and granulopoesis factors, all of which contribute to the cutaneous host defense against *S. aureus*.

Keratinocytes express pattern recognition receptors such as TLR2, which recognizes *S. aureus* lipopeptides and lipoteichoic acid, and nucleotide-binding oligomerization domain containing 2 (NOD2), which recognizes the *S. aureus* peptidoglycan breakdown product muramyl dipeptide. Both the TLR2 and NOD2 signals lead to activation of nuclear factor-κB (NF-κB) and other transcription factors that induce transcription of the proinflammatory mediators (cytokines, chemokines, adhesion molecules and AMPs) involved in cutaneous host defense against *S. aureus* (Figure 1) [102,103]. Upon cutaneous *S. aureus* infection, the epidermal barrier is breached and keratinocytes and resident skin immune cells (e.g., Langerhans cells and γδ T cells in the epidermis, as well as dendritic cells, macrophages, fibroblasts, mast cells, B and T cells, plasma cells and natural killer cells in the dermis) produce pro-inflammatory cytokines, chemokines and adhesion molecules. These molecules promote the recruitment of neutrophils from the bloodstream, which help to control the infection by forming an abscess. Neutrophilic abscess formation is a hallmark of *S. aureus* infections, which are typically pyogenic, and is required for bacterial clearance. Pro-inflammatory cytokines also induce the production of AMPs (e.g., β-defensins and cathelicidins) with bacteriostatic or bactericidal activity against *S. aureus* [104,105].

## 6. Cutaneous Host Defense Involving IL-1- and IL-17-Mediated AMP Activity against *S. aureus*

**Figure 2.**
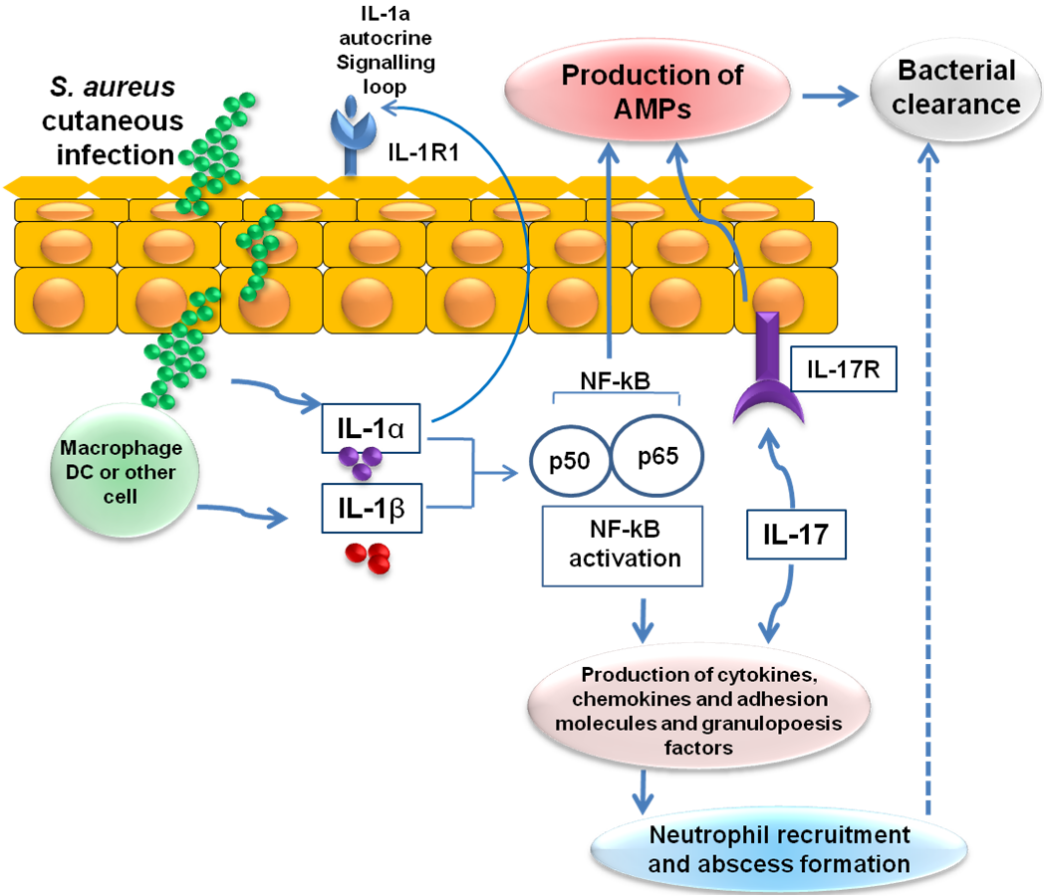
IL-1- and IL-17-mediated cutaneous immune response against *S. aureus*. Infection of the skin by *S. aureus* leads to the production of IL-1α, IL-1β and IL-17, which in turn triggers activation of nuclear factor-κB (NF-κB). These signaling pathways lead to the production of AMPs, cytokines, chemokines, adhesion molecules and granulopoesis factors, which recruit neutrophils from the circulation to the site of *S. aureus* infection in the skin. The recruited neutrophils form an abscess that helps control and limit the spread of the infection, and is ultimately required for bacterial clearance. IL-1R1, interleukin-1 receptor 1; IL-17R, interleukin-17 receptor.

IL-1α, which is produced and released by keratinocytes, and IL-1β, which is produced by resident and recruited immune cells (e.g., macrophages and dendritic cells), trigger activation of NF-κB. These signaling pathways lead to the production of β-defensins 2 and 3, cathelicidins and RNase 7. IL-1-mediated responses also result in the production of pro-inflammatory cytokines, chemokines and adhesion molecules that promote the recruitment of neutrophils from the circulation to sites of *S. aureus* infection in the skin and abscess formation (Figure 2) [106]. In addition to IL-1, recent studies have uncovered the critical role played by IL-17, which is predominantly expressed by recruited T cell subsets (Th17 cells, NKT cells and γδ T cells) and natural killer cells in response to TLR2 activation. The IL-17 produced stimulates production of β-defensins 2 and 3 and cathelicidins by keratinocytes and induces neutrophil recruitment via induction of various chemokines (CXCL1, CXCL2 and IL-8) and granulopoesis factors (G-CSF and GM-CSF) [107].

## 7. Mechanisms by Which *S. aureus* Evades Skin-Derived CAMPs

**Figure 3.**
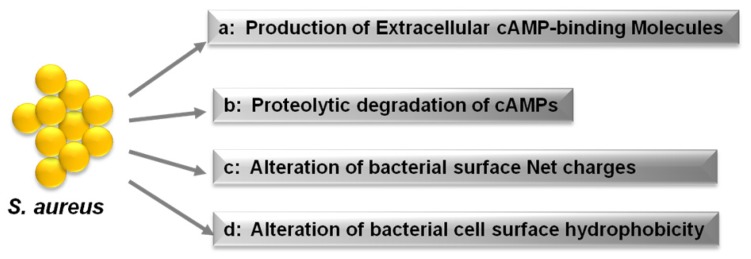
Strategies by which *S. aureus* evades CAMPs. *S. aureus* counteracts CAMPs by secreting trapping molecules and proteases that inactivate CAMPs and by modifying the cell membrane hydrophobicity or net charge [108].

The importance of CAMPs in host cutaneous defense against *S. aureus* is evidenced by the mechanisms that have evolved in *S. aureus* to resist and evade these peptides. As shown in Figure 3, *S. aureus* counteracts CAMPs and antimicrobial fatty acids through at least four mechanisms: (i) production of CAMP-binding molecules, like the fibrinolytic enzyme staphylokinase (SAK), which binds to and inhibits α-defensins; (ii) proteolytic degradation of CAMPs by secreted proteases such as aureolysin, which cleaves and inactivates LL-37; (iii) reduction of the bacterial cell surface net negative charge by modification of teichoic acids using d-alanine or phospholipids with l-lysine; and (iv) alteration of bacterial cell surface hydrophobicity [11,108]. In the following sections, these mechanisms will be discussed in additional detail.

### 7.1. Secretion of Extracellular CAMP-Binding Molecules

*S. aureus* resists α-defensins through the production of SAK, which binds human α-defensins with high affinity, thereby mediating significant α-defensin resistance. *In vitro*, SAK levels correlate inversely with the susceptibility of *S. aureus* isolates to α-defensins (Figure 3a) [70].

### 7.2. Proteolytic Degradation of CAMPs by Secreted Proteases

*S. aureus* and many other bacterial species produce peptidases and proteases capable of cleaving CAMPs. *In vitro*, production of *S. aureus* protease correlates with staphylococcal resistance to CAMPs [36]. For example, *S. aureus* produces a metalloproteinase, aureolysin, which cleaves and inactivates LL-37 [36]. *S. aureus* also secretes extracellular proteases that degrade dermcidin, neutralizing its antimicrobial activity [84], and similar observations have been made with the *S. epidermidis* protease SepA (Figure 3b) [84].

### 7.3. Resistance to CAMPs through Reduction of Bacterial Surface Net Negative Charges

CAMPs and most other antimicrobial molecules, including lysozyme, phospholipase A2 and RNase5, have net positive charges. The surface of human cells is normally composed mainly of uncharged or zwitterionic lipids, whereas bacterial surfaces are composed of various anionic components, including as peptidoglycan, the phospholipids phosphatidylglycerol and cardiolipin, lipid A, and teichoic acids, which give it an anionic net charge [109]. Presumably, antimicrobial host factors have evolved to be cationic to achieve strong, selective affinity for the anionic surfaces of bacteria. However, some bacterial species, like *S. aureus*, are able to reduce the negative charge of their cell envelope, thereby becoming resistant to inactivation by many CAMPs (Figure 3c) [69,71–73,110–112].

#### 7.3.1. Modification of Phospholipids with l l -lysine

To reduce anionic charge, *S. aureus* and other bacteria are able to modify most of their phosphatidylglycerol with l-lysine [113]. The lysinylation of phosphatidylglycerol is mediated by a membrane protein, multiple peptide resistance factor protein (MprF) [112,114], which neutralizes the bacterial cell envelope and thus reduces susceptibility to many CAMPs, including α-defensins, LL-37 and Group IIA-phospholipase A_2_ [69,71,110,111].

#### 7.3.2. Modification of Teichoic Acids with d-alanine

Products of the *dlt*ABCD operon attach positively charged d-alanine residues to negatively charged phosphate groups in the backbone of teichoic acids, rendering bacteria less susceptible to α-defensins and LL-37 [72,73]. Teichoic acids in *S. aureus* and other Gram-positive bacteria consist of alternating glycerolphosphate or ribitolphosphate units, which are substituted with *N*-acetyl-glucosamine or d-alanine [113]. These polymers are either anchored to the cytoplasmic membrane (lipoteichoic acid) or to the peptidoglycan cell wall (wall teichoic acid) and are anionic due to the presence of negatively charged phosphate groups. In a fashion similar to lysinylation of phospholipids, modification of teichoic acids with d-alanine leads to a partial neutralization of the polymer [73], which reduces the interaction of CAMPs with the bacterial surface, in turn reducing the susceptibility to cationic host defense molecules, including α-defensins and LL-37 [73]. Consistent with this scenario, an *S. aureus* mutant lacking d-alanine in its teichoic acids (*dlt*A mutant) as well as clinical isolates expressing lower levels of *dlt*A showed greater susceptibility to CAMPs, including dermcidin, RNase 7, hBD2 and hBD3 [76]. With dermcidin, cationic structures in the *N*-terminal part of the peptide appear crucial for interaction with the negative bacterial cell surface, which likely explains why d-alanylation influences its efficacy [83]. Also, several studies in animal models have demonstrated that alanylated teichoic acids contribute to an increased virulence of *S. aureus* [115–117].

### 7.4. Resistance to CAMPs through Alteration of Bacterial Cell Surface Hydrophobicity

Human skin is rich in antimicrobial fatty acids produced by sebaceous glands. *S. aureus* produces IsdA, which alters its surface hydrophobicity, thereby reducing the efficiency with which fatty acids gain access to the cells [35]. Indeed, by decreasing cellular hydrophobicity, IsdA renders *S. aureus* resistant to hBD2 and LL-37 on human skin (Figure 3d) [35].

## 8. Conclusions

*S. aureus* is a frequent component of human skin and nose microbiota. However, it can also cause various skin diseases, sometimes leading to systemic infections. The ability of *S. aureus* to colonize and infect the skin is apparently dependent on specific mechanisms that subvert host cutaneous defenses. The existence of these multiple resistance mechanisms makes it clear that CAMPs play a key role in the host cutaneous defense against *S. aureus*. Peschel *et al.* proposed the coevolution of CAMP structures and bacterial resistance mechanisms, which has led to the existence of the currently observed CAMPs [118]. One mechanism that renders CAMPs resistant to degradation by proteases is stabilization through disulphide bridges [118]. Other modifications that increase the efficacy of CAMPs include variation in the amino acid sequence, increases in the net positive charge through incorporation of larger numbers of cationic amino acids, and combining multiple antimicrobial mechanisms in a single molecule. These potential modifications of CAMPs are reviewed in detail by Peschel and Sahl [118].

Ouhara *et al.* showed that several clinical isolates of MRSA strains exhibited reduced susceptibility to the human LL-37 but not to the hBD3 [119]. The greater resistance to LL-37 is based on the more positive net cell-surface charge in MRSA strains than methicillin-susceptible *S. aureus* strains (MSSA). The fact that the efficacy of hBD3 appears unaffected may be due to its more positive net charge, as compared to LL-37 (+11 *vs.* +6), which would favor stronger interaction with the bacterial cell surface [119]. This suggests targeting highly conserved bacterial CAMP resistance mechanisms such as lysinylation of phospholipids by MprF or the alanylation of teichoic acids by *dlt*ABCD could be an effective treatment strategy. Moreover, in addition to its essential role in mediating resistance to CAMPs [69,111], it appears MprF may dramatically reduce the susceptibility of *S. aureus* to the novel lipopeptide antibiotic daptomycin [120,121]. Thus, new therapeutics targeting CAMP resistance factors, like MprF, could not only render a pathogen susceptible to host antimicrobial defense, but might also act synergistically to combat infections in combination with currently available antibiotics. Although targeting resistance factors would not directly inactivate *S. aureus*, it would render it more susceptible to CAMPs, thus assisting host cutaneous defenses to successfully combat skin infections.

The increasing numbers of reports of virulent and drug-resistant strains of *S. aureus* prompt further investigation into the mechanisms that enable this pathogen to cause infection and overcome the broad spectrum of human cutaneous antimicrobial defenses. We anticipate that future studies will provide further information about the host and bacterial determinants involved in skin colonization and infection by *S. aureus*. Targeted drug development around highly conserved bacterial resistance mechanisms against host CAMPs is a promising pharmacologic approach in this era of highly virulent and drug-resistant strains of *S. aureus*.

## Figures and Tables

**Table 1.** Cationic antimicrobial peptides (CAMPs) that contribute to human cutaneous immune defenses against *S. aureus*.

Peptides	Cellular source in the skin	Mechanism of *S. aureus* evasion	References
α-Defensins	Neutrophils	Staphylokinase, MprF, *dlt*ABCD operon	[67–73]
hBD2	Keratinocytes, macrophages, and dendritic cells	IsdA, *dlt*ABCD	[26,29,35,74–76]
hBD3	Keratinocytes	*dlt*ABCD operon	[23,24,27,75–80]
hBD4	Keratinocytes	Please check	[81]
LL-37	Keratinocytes, macrophages, and neutrophils	IsdA, Aureolysin, MprF, *dlt*ABCD	[25,29,35,36,69,71–73,75]
Dermcidin	Sweat glands	Extracellular proteases, *dlt*ABCD operon	[82–84]
RNase 7	Keratinocytes	*dlt*ABCD operon	[24,28,61,76]

## References

[1] Chambers H.F. (2001). The changing epidemiology of *Staphylococcus aureus*?. Emerg. Infect. Dis.

[2] Lowy F.D. (1998). *Staphylococcus aureus* infections. N. Engl. J. Med.

[3] Graham P.L., Lin S. X., Larson E.L. (2006). A U.S. population-based survey of *Staphylococcus aureus* colonization. Ann. Intern. Med.

[4] Kuehnert M.J., Kruszon-Moran D., Hill H.A., McQuillan G., McAllister S.K., Fosheim G., McDougal L.K., Chaitram J., Jensen B., Fridkin S.K. (2006). Prevalence of *Staphylococcus aureus* nasal colonization in the United States, 2001–2002. J. Infect. Dis.

[5] Perl T.M., Cullen J.J., Wenzel R.P., Zimmerman M.B., Pfaller M.A., Sheppard D., Twombley J., French P.P., Herwaldt L.A. (2002). Intranasal mupirocin to prevent postoperative *Staphylococcus aureus* infections. N. Engl. J. Med.

[6] Shopsin B., Mathema B., Martinez J., Ha E., Campo M.L., Fierman A., Krasinski K., Kornblum J., Alcabes P., Waddington M., Riehman M. (2000). Prevalence of methicillin-resistant and methicillin-susceptible *Staphylococcus aureus* in the community. J. Infect. Dis.

[7] Von Eiff C., Becker K., Machka K., Stammer H., Peters G. (2001). Nasal carriage as a source of *Staphylococcus aureus* bacteremia. N. Engl. J. Med.

[8] Herwaldt L.A. (2003). *Staphylococcus aureus* nasal carriage and surgical-site infections. Surgery.

[9] Lin Y.C., Lauderdale T.L., Lin H.M., Chen P.C., Cheng M.F., Hsieh K.S., Liu Y.C. (2007). An outbreak of methicillin-resistant *Staphylococcus aureus* infection in patients of a pediatric intensive care unit and high carriage rate among health care workers. J. Microbiol. Immunol. Infect.

[10] Elston D.M. (2007). Community-acquired methicillin-resistant *Staphylococcus aureus*. J. Am. Acad. Dermatol.

[11] Foster T.J. (2005). Immune evasion by staphylococci. Nat. Rev. Microbiol.

[12] Kraus D., Peschel A. (2006). Molecular mechanisms of bacterial resistance to antimicrobial peptides. Curr. Top. Microbiol. Immunol.

[13] Glaser R., Becker K., von Eiff C., Meyer-Hoffert U., Harder J. (2014). Decreased susceptibility of Staphylococcus aureus small colony variants (SCVs) towards human antimicrobial peptides. J. Invest. Dermatol.

[14] Grenfell B.T., Pybus O.G., Gog J.R., Wood J.L., Daly J.M., Mumford J.A., Holmes E.C. (2004). Unifying the epidemiological and evolutionary dynamics of pathogens. Science.

[15] Woolhouse M.E., Webster J.P., Domingo E., Charlesworth B., Levin B.R. (2002). Biological and biomedical implications of the co-evolution of pathogens and their hosts. Nat. Genet.

[16] Gorwitz R.J., Kruszon-Moran D., McAllister S.K., McQuillan G., McDougal L.K., Fosheim G.E., Jensen B.J., Killgore G., Tenover F.C., Kuehnert M.J. (2008). Changes in the prevalence of nasal colonization with *Staphylococcus aureus* in the United States, 2001–2004. J. Infect. Dis.

[17] Miller L.G., Diep B.A. (2008). Clinical practice: Colonization, fomites, and virulence: Rethinking the pathogenesis of community-associated methicillin-resistant *Staphylococcus aureus* infection. Clin. Infect. Dis.

[18] Grice E.A., Kong H.H., Conlan S., Deming C.B., Davis J., Young A.C., Program N.C.S., Bouffard G.G., Blakesley R.W., Murray P.R. (2009). Topographical and temporal diversity of the human skin microbiome. Science.

[19] Grice E.A., Segre J.A. (2011). The skin microbiome. Nat. Rev. Microbiol.

[20] Miajlovic H., Fallon P.G., Irvine A.D., Foster T.J. (2010). Effect of filaggrin breakdown products on growth of and protein expression by *Staphylococcus aureus*. J. Allergy. Clin. Immunol.

[21] Iwase T., Uehara Y., Shinji H., Tajima A., Seo H., Takada K., Agata T., Mizunoe Y. (2010). *Staphylococcus epidermidis* Esp inhibits *Staphylococcus aureus* biofilm formation and nasal colonization. Nature.

[22] Cogen A.L., Yamasaki K., Sanchez K.M., Dorschner R.A., Lai Y., MacLeod D.T., Torpey J.W., Otto M., Nizet V., Kim J.E. (2010). Selective antimicrobial action is provided by phenol-soluble modulins derived from *Staphylococcus epidermidis*, a normal resident of the skin. J. Invest. Dermatol.

[23] Lai Y., Cogen A.L., Radek K.A., Park H.J., Macleod D.T., Leichtle A., Ryan A.F., Di Nardo A., Gallo R.L. (2010). Activation of TLR2 by a small molecule produced by *Staphylococcus epidermidis* increases antimicrobial defense against bacterial skin infections. J. Invest. Dermatol.

[24] Wanke I., Steffen H., Christ C., Krismer B., Gotz F., Peschel A., Schaller M., Schittek B. (2011). Skin commensals amplify the innate immune response to pathogens by activation of distinct signaling pathways. J. Invest. Dermatol.

[25] Braff M.H., Zaiou M., Fierer J., Nizet V., Gallo R.L. (2005). Keratinocyte production of cathelicidin provides direct activity against bacterial skin pathogens. Infect. Immun.

[26] Harder J., Bartels J., Christophers E., Schroder J.M. (1997). A peptide antibiotic from human skin. Nature.

[27] Kisich K.O., Howell M.D., Boguniewicz M., Heizer H.R., Watson N.U., Leung D.Y. (2007). The constitutive capacity of human keratinocytes to kill *Staphylococcus aureus* is dependent on β-defensin 3. J. Invest. Dermatol.

[28] Simanski M., Dressel S., Glaser R., Harder J. (2010). RNase 7 protects healthy skin from *Staphylococcus aureus* colonization. J. Invest. Dermatol.

[29] Ong P.Y., Ohtake T., Brandt C., Strickland I., Boguniewicz M., Ganz T., Gallo R.L., Leung D.Y. (2002). Endogenous antimicrobial peptides and skin infections in atopic dermatitis. N. Engl. J. Med.

[30] Burian M., Rautenberg M., Kohler T., Fritz M., Krismer B., Unger C., Hoffmann W.H., Peschel A., Wolz C., Goerke C. (2010). Temporal expression of adhesion factors and activity of global regulators during establishment of *Staphylococcus aureus* nasal colonization. J. Infect. Dis.

[31] Cho S.H., Strickland I., Tomkinson A., Fehringer A.P., Gelfand E.W., Leung D.Y. (2001). Preferential binding of *Staphylococcus aureus* to skin sites of Th2-mediated inflammation in a murine model. J. Invest. Dermatol.

[32] Clarke S.R., Brummell K.J., Horsburgh M.J., McDowell P.W., Mohamad S.A., Stapleton M.R., Acevedo J., Read R.C., Day N.P., Peacock S.J. (2006). Identification of *in vivo*-expressed antigens of *Staphylococcus aureus* and their use in vaccinations for protection against nasal carriage. J. Infect. Dis.

[33] Weidenmaier C., Kokai-Kun J.F., Kristian S.A., Chanturiya T., Kalbacher H., Gross M., Nicholson G., Neumeister B., Mond J.J., Peschel A. (2004). Role of teichoic acids in *Staphylococcus aureus* nasal colonization, a major risk factor in nosocomial infections. Nat. Med.

[34] Laouini D., Kawamoto S., Yalcindag A., Bryce P., Mizoguchi E., Oettgen H., Geha R.S. (2003). Epicutaneous sensitization with superantigen induces allergic skin inflammation. J. Allergy. Clin. Immunol.

[35] Clarke S.R., Mohamed R., Bian L., Routh A.F., Kokai-Kun J.F., Mond J.J., Tarkowski A., Foster S.J. (2007). The *Staphylococcus aureus* surface protein IsdA mediates resistance to innate defenses of human skin. Cell Host Microbe.

[36] Sieprawska-Lupa M., Mydel P., Krawczyk K., Wojcik K., Puklo M., Lupa B., Suder P., Silberring J., Reed M., Pohl J. (2004). Degradation of human antimicrobial peptide LL-37 by *Staphylococcus aureus*-derived proteinases. Antimicrob. Agents Chemother.

[37] Zecconi A., Scali F. (2013). *Staphylococcus aureus* virulence factors in evasion from innate immune defenses in human and animal diseases. Immunol. Lett.

[38] Hallander H.O., Laurell G., Lofstrom G. (1966). Enhancement of staphylococcal pathogenicity in the presence of penicillin. Acta Pathol. Microbiol. Scand.

[39] Ohlsen K., Ziebuhr W., Koller K.P., Hell W., Wichelhaus T.A., Hacker J. (1998). Effects of subinhibitory concentrations of antibiotics on α-toxin (*hla*) gene expression of methicillin-sensitive and methicillin-resistant *Staphylococcus aureus* isolates. Antimicrob. Agents Chemother.

[40] Shibl A.M. (1983). Effect of antibiotics on production of enzymes and toxins by microorganisms. Rev. Infect. Dis.

[41] Bernardo K., Pakulat N., Fleer S., Schnaith A., Utermohlen O., Krut O., Muller S., Kronke M. (2004). Subinhibitory concentrations of linezolid reduce *Staphylococcus aureus* virulence factor expression. Antimicrob. Agents Chemother.

[42] Schlievert P.M., Kelly J.A. (1984). Clindamycin-induced suppression of toxic-shock syndrome— Associated exotoxin production. J. Infect. Dis.

[43] Shibl A.M. (1981). Role of *Staphylococcus aureus* exfoliatin toxin in staphylococcal infections in mice. Chemotherapy.

[44] Veringa E. M., Verhoef J. (1986). Influence of subinhibitory concentrations of clindamycin on opsonophagocytosis of *Staphylococcus aureus*, a protein-A-dependent process. Antimicrob. Agents Chemother.

[45] Beilman G.J., Sandifer G., Skarda D., Jensen B., McAllister S., Killgore G., Srinivasan A. (2005). Emerging infections with community-associated methicillin-resistant *Staphylococcus aureus* in outpatients at an Army Community Hospital. Surg. Infect. (Larchmt).

[46] Daum R.S. (2007). Clinical practice: Skin and soft-tissue infections caused by methicillin-resistant *Staphylococcus aureus*. N. Engl. J. Med.

[47] David M.Z., Daum R.S. (2010). Community-associated methicillin-resistant *Staphylococcus aureus*: Epidemiology and clinical consequences of an emerging epidemic. Clin. Microbiol. Rev.

[48] DeLeo F.R., Otto M., Kreiswirth B.N., Chambers H.F. (2010). Community-associated meticillin-resistant *Staphylococcus aureus*. Lancet.

[49] Deurenberg R.H., Stobberingh E.E. (2008). The evolution of *Staphylococcus aureus*. Infect. Genet. Evol.

[50] Mediavilla J.R., Chen L., Mathema B., Kreiswirth B.N. (2012). Global epidemiology of community-associated methicillin resistant *Staphylococcus aureus* (CA-MRSA). Curr. Opin. Microbiol.

[51] Chavez T.T., Decker C.F. (2008). Health care-associated MRSA *versus* community-associated MRSA. Dis. Mon.

[52] Enright M.C. (2003). The evolution of a resistant pathogen—The case of MRSA. Curr. Opin. Pharmacol.

[53] Naimi T.S., LeDell K.H., Como-Sabetti K., Borchardt S.M., Boxrud D.J., Etienne J., Johnson S.K., Vandenesch F., Fridkin S., O’Boyle C. (2003). Comparison of community- and health care-associated methicillin-resistant *Staphylococcus aureus* infection. J. Am. Med. Assoc.

[54] Klein E., Smith D.L., Laxminarayan R. (2009). Community-associated methicillin-resistant *Staphylococcus aureus* in outpatients, United States, 1999–2006. Emerg. Infect. Dis.

[55] Ghuysen J.M. (1994). Molecular structures of penicillin-binding proteins and β-lactamases. Trends. Microbiol.

[56] Katayama Y., Ito T., Hiramatsu K. (2000). A new class of genetic element, staphylococcus cassette chromosome *mec*, encodes methicillin resistance in *Staphylococcus aureus*. Antimicrob. Agents Chemother.

[57] Hiramatsu K., Cui L., Kuroda M., Ito T. (2001). The emergence and evolution of methicillin-resistant *Staphylococcus aureus*. Trends. Microbiol.

[58] Diekema D.J., Pfaller M.A., Schmitz F.J., Smayevsky J., Bell J., Jones R.N., Beach M., Group S.P. (2001). Survey of infections due to *Staphylococcus* species: Frequency of occurrence and antimicrobial susceptibility of isolates collected in the United States, Canada, Latin America, Europe, and the Western Pacific region for the SENTRY Antimicrobial Surveillance Program, 1997–1999. Clin. Infect. Dis.

[59] Centers for Disease Control and Prevention (2002). *Staphylococcus aureus* resistant to vancomycin—United States, 2002. MMWR Morb. Mortal. Wkly. Rep.

[60] Hiramatsu K., Hanaki H., Ino T., Yabuta K., Oguri T., Tenover F.C. (1997). Methicillin-resistant *Staphylococcus aureus* clinical strain with reduced vancomycin susceptibility. J. Antimicrob. Chemother.

[61] Cho J.S., Xuan C., Miller L.S. (2010). Lucky number seven: RNase 7 can prevent *Staphylococcus aureus* skin colonization. J. Invest. Dermatol.

[62] Otto M. (2010). Staphylococcus colonization of the skin and antimicrobial peptides. Expert. Rev. Dermatol.

[63] Schauber J., Gallo R.L. (2009). Antimicrobial peptides and the skin immune defense system. J. Allergy. Clin. Immunol.

[64] Sahl H.G., Pag U., Bonness S., Wagner S., Antcheva N., Tossi A. (2005). Mammalian defensins: Structures and mechanism of antibiotic activity. J. Leukoc. Biol.

[65] Miller L.S., Sorensen O.E., Liu P.T., Jalian H.R., Eshtiaghpour D., Behmanesh B.E., Chung W., Starner T.D., Kim J., Sieling P.A. (2005). TGF-α regulates TLR expression and function on epidermal keratinocytes. J. Immunol.

[66] Sorensen O.E., Thapa D.R., Roupe K.M., Valore E.V., Sjobring U., Roberts A.A., Schmidtchen A., Ganz T. (2006). Injury-induced innate immune response in human skin mediated by transactivation of the epidermal growth factor receptor. J. Clin. Invest.

[67] Lehrer R.I. (2007). Multispecific myeloid defensins. Curr. Opin. Hematol.

[68] Ericksen B., Wu Z., Lu W., Lehrer R.I. (2005). Antibacterial activity and specificity of the six human α-defensins. Antimicrob. Agents Chemother.

[69] Peschel A., Jack R.W., Otto M., Collins L.V., Staubitz P., Nicholson G., Kalbacher H., Nieuwenhuizen W.F., Jung G., Tarkowski A. (2001). *Staphylococcus aureus* resistance to human defensins and evasion of neutrophil killing via the novel virulence factor MprF is based on modification of membrane lipids with l-lysine. J. Exp. Med.

[70] Jin T., Bokarewa M., Foster T., Mitchell J., Higgins J., Tarkowski A. (2004). *Staphylococcus aureus* resists human defensins by production of staphylokinase, a novel bacterial evasion mechanism. J. Immunol.

[71] Ernst C.M., Staubitz P., Mishra N.N., Yang S.J., Hornig G., Kalbacher H., Bayer A.S., Kraus D., Peschel A. (2009). The bacterial defensin resistance protein MprF consists of separable domains for lipid lysinylation and antimicrobial peptide repulsion. PLoS Pathog.

[72] Jann N.J., Schmaler M., Kristian S.A., Radek K.A., Gallo R.L., Nizet V., Peschel A., Landmann R. (2009). Neutrophil antimicrobial defense against *Staphylococcus aureus* is mediated by phagolysosomal but not extracellular trap-associated cathelicidin. J. Leukoc. Biol.

[73] Peschel A., Otto M., Jack R.W., Kalbacher H., Jung G., Gotz F. (1999). Inactivation of the dlt operon in *Staphylococcus aureus* confers sensitivity to defensins, protegrins, and other antimicrobial peptides. J. Biol. Chem.

[74] Dinulos J.G., Mentele L., Fredericks L.P., Dale B.A., Darmstadt G.L. (2003). Keratinocyte expression of human β-defensin 2 following bacterial infection: Role in cutaneous host defense. Clin. Diagn. Lab. Immunol.

[75] Sayama K., Komatsuzawa H., Yamasaki K., Shirakata Y., Hanakawa Y., Ouhara K., Tokumaru S., Dai X., Tohyama M., Ten Dijke P. (2005). New mechanisms of skin innate immunity: ASK1-mediated keratinocyte differentiation regulates the expression of β-defensins, LL37, and TLR2. Eur. J. Immunol.

[76] Simanski M., Glaser R., Koten B., Meyer-Hoffert U., Wanner S., Weidenmaier C., Peschel A., Harder J. (2013). *Staphylococcus aureus* subverts cutaneous defense by d-alanylation of teichoic acids. Exp. Dermatol.

[77] Harder J., Bartels J., Christophers E., Schroder J.M. (2001). Isolation and characterization of human β-defensin 3, a novel human inducible peptide antibiotic. J. Biol. Chem.

[78] Menzies B.E., Kenoyer A. (2006). Signal transduction and nuclear responses in *Staphylococcus aureus*-induced expression of human β-defensin 3 in skin keratinocytes. Infect. Immun.

[79] Sumikawa Y., Asada H., Hoshino K., Azukizawa H., Katayama I., Akira S., Itami S. (2006). Induction of β-defensin 3 in keratinocytes stimulated by bacterial lipopeptides through Toll-like receptor 2. Microbes. Infect.

[80] Zanger P., Holzer J., Schleucher R., Scherbaum H., Schittek B., Gabrysch S. (2010). Severity of *Staphylococcus aureus* infection of the skin is associated with inducibility of human β-defensin 3 but not human β-defensin 2. Infect. Immun.

[81] Garcia J.R., Krause A., Schulz S., Rodriguez-Jimenez F.J., Kluver E., Adermann K., Forssmann U., Frimpong-Boateng A., Bals R., Forssmann W.G. (2001). Human β-defensin 4: A novel inducible peptide with a specific salt-sensitive spectrum of antimicrobial activity. FASEB J.

[82] Rieg S., Steffen H., Seeber S., Humeny A., Kalbacher H., Dietz K., Garbe C., Schittek B. (2005). Deficiency of dermcidin-derived antimicrobial peptides in sweat of patients with atopic dermatitis correlates with an impaired innate defense of human skin *in vivo*. J. Immunol..

[83] Steffen H., Rieg S., Wiedemann I., Kalbacher H., Deeg M., Sahl H.G., Peschel A., Gotz F., Garbe C., Schittek B. (2006). Naturally processed dermcidin-derived peptides do not permeabilize bacterial membranes and kill microorganisms irrespective of their charge. Antimicrob. Agents Chemother.

[84] Lai Y., Villaruz A.E., Li M., Cha D.J., Sturdevant D.E., Otto M. (2007). The human anionic antimicrobial peptide dermcidin induces proteolytic defence mechanisms in staphylococci. Mol. Microbiol.

[85] Grigat J., Soruri A., Forssmann U., Riggert J., Zwirner J. (2007). Chemoattraction of macrophages, T lymphocytes, and mast cells is evolutionarily conserved within the human α-defensin family. J. Immunol.

[86] Rohrl J., Yang D., Oppenheim J.J., Hehlgans T. (2010). Human β-defensin 2 and 3 and their mouse orthologs induce chemotaxis through interaction with CCR2. J. Immunol.

[87] Yang D., Chertov O., Bykovskaia S.N., Chen Q., Buffo M.J., Shogan J., Anderson M., Schroder J.M., Wang J.M., Howard O.M. (1999). β-Defensins: Linking innate and adaptive immunity through dendritic and T cell CCR6. Science.

[88] De Y., Chen Q., Schmidt A.P., Anderson G.M., Wang J.M., Wooters J., Oppenheim J.J., Chertov O. (2000). LL-37, the neutrophil granule- and epithelial cell-derived cathelicidin, utilizes formyl peptide receptor-like 1 (FPRL1) as a receptor to chemoattract human peripheral blood neutrophils, monocytes, and T cells. J. Exp. Med.

[89] Tjabringa G.S., Ninaber D.K., Drijfhout J.W., Rabe K.F., Hiemstra P.S. (2006). Human cathelicidin LL-37 is a chemoattractant for eosinophils and neutrophils that acts via formyl-peptide receptors. Int. Arch. Allergy. Immunol.

[90] Ganz T. (2003). Defensins: Antimicrobial peptides of innate immunity. Nat. Rev. Immunol.

[91] Harder J., Meyer-Hoffert U., Wehkamp K., Schwichtenberg L., Schroder J.M. (2004). Differential gene induction of human β-defensins (hBD-1, -2, -3, and -4) in keratinocytes is inhibited by retinoic acid. J. Invest. Dermatol.

[92] Miller L.S., Modlin R.L. (2007). Human keratinocyte Toll-like receptors promote distinct immune responses. J. Invest. Dermatol.

[93] Yang D., Chertov O., Oppenheim J.J. (2001). Participation of mammalian defensins and cathelicidins in anti-microbial immunity: Receptors and activities of human defensins and cathelicidin (LL-37). J. Leukoc. Biol.

[94] Zanetti M. (2004). Cathelicidins, multifunctional peptides of the innate immunity. J. Leukoc. Biol.

[95] Murakami M., Ohtake T., Dorschner R.A., Schittek B., Garbe C., Gallo R.L. (2002). Cathelicidin anti-microbial peptide expression in sweat, an innate defense system for the skin. J. Invest. Dermatol.

[96] Liu P.T., Stenger S., Li H., Wenzel L., Tan B.H., Krutzik S.R., Ochoa M.T., Schauber J., Wu K., Meinken C. (2006). Toll-like receptor triggering of a vitamin D-mediated human antimicrobial response. Science.

[97] Schauber J., Dorschner R.A., Coda A.B., Buchau A.S., Liu P.T., Kiken D., Helfrich Y.R., Kang S., Elalieh H.Z., Steinmeyer A. (2007). Injury enhances TLR2 function and antimicrobial peptide expression through a vitamin D-dependent mechanism. J. Clin. Invest.

[98] Wang T.T., Nestel F.P., Bourdeau V., Nagai Y., Wang Q., Liao J., Tavera-Mendoza L., Lin R., Hanrahan J.W., Mader S. (2004). Cutting edge: 1,25-Dihydroxyvitamin D3 is a direct inducer of antimicrobial peptide gene expression. J. Immunol.

[99] Zhang J., Dyer K.D., Rosenberg H.F. (2003). Human RNase 7: A new cationic ribonuclease of the RNase A superfamily. Nucleic. Acids. Res.

[100] Rieg S., Garbe C., Sauer B., Kalbacher H., Schittek B. (2004). Dermcidin is constitutively produced by eccrine sweat glands and is not induced in epidermal cells under inflammatory skin conditions. Br. J. Dermatol.

[101] Schittek B., Hipfel R., Sauer B., Bauer J., Kalbacher H., Stevanovic S., Schirle M., Schroeder K., Blin N., Meier F. (2001). Dermcidin: A novel human antibiotic peptide secreted by sweat glands. Nat. Immunol.

[102] Hruz P., Zinkernagel A.S., Jenikova G., Botwin G.J., Hugot J.P., Karin M., Nizet V., Eckmann L. (2009). NOD2 contributes to cutaneous defense against *Staphylococcus aureus* through α-toxin-dependent innate immune activation. Proc. Natl. Acad. Sci. USA.

[103] Takeuchi O., Akira S. (2010). Pattern recognition receptors and inflammation. Cell.

[104] Kim M.H., Granick J.L., Kwok C., Walker N.J., Borjesson D.L., Curry F.R., Miller L.S., Simon S.I. (2011). Neutrophil survival and c-kit^+^-progenitor proliferation in *Staphylococcus aureus*-infected skin wounds promote resolution. Blood.

[105] Molne L., Verdrengh M., Tarkowski A. (2000). Role of neutrophil leukocytes in cutaneous infection caused by *Staphylococcus aureus*. Infect. Immun.

[106] Miller L.S., Cho J.S. (2011). Immunity against *Staphylococcus aureus* cutaneous infections. Nat. Rev. Immunol.

[107] Cua D.J., Tato C.M. (2010). Innate IL-17-producing cells: The sentinels of the immune system. Nat. Rev. Immunol.

[108] Kraus D., Peschel A. (2008). *Staphylococcus aureus* evasion of innate antimicrobial defense. Future Microbiol.

[109] Huijbregts R.P., de Kroon A.I., de Kruijff B. (2000). Topology and transport of membrane lipids in bacteria. Biochim. Biophys. Acta.

[110] Koprivnjak T., Peschel A., Gelb M.H., Liang N.S., Weiss J.P. (2002). Role of charge properties of bacterial envelope in bactericidal action of human group IIA phospholipase A2 against *Staphylococcus aureus*. J. Biol. Chem.

[111] Kristian S.A., Durr M., van Strijp J.A., Neumeister B., Peschel A. (2003). MprF-mediated lysinylation of phospholipids in *Staphylococcus aureus* leads to protection against oxygen-independent neutrophil killing. Infect. Immun.

[112] Staubitz P., Neumann H., Schneider T., Wiedemann I., Peschel A. (2004). MprF-mediated biosynthesis of lysylphosphatidylglycerol, an important determinant in staphylococcal defensin resistance. FEMS Microbiol. Lett.

[113] Neuhaus F.C., Baddiley J. (2003). A continuum of anionic charge: Structures and functions of d-alanyl-teichoic acids in gram-positive bacteria. Microbiol. Mol. Biol. Rev.

[114] Oku Y., Kurokawa K., Ichihashi N., Sekimizu K. (2004). Characterization of the *Staphylococcus aureus mprF* gene, involved in lysinylation of phosphatidylglycerol. Microbiology.

[115] Collins L.V., Kristian S.A., Weidenmaier C., Faigle M., van Kessel K.P., van Strijp J.A., Gotz F., Neumeister B., Peschel A. (2002). *Staphylococcus aureus* strains lacking d-alanine modifications of teichoic acids are highly susceptible to human neutrophil killing and are virulence attenuated in mice. J. Infect. Dis.

[116] Kristian S.A., Lauth X., Nizet V., Goetz F., Neumeister B., Peschel A., Landmann R. (2003). Alanylation of teichoic acids protects *Staphylococcus aureus* against Toll-like receptor 2-dependent host defense in a mouse tissue cage infection model. J. Infect. Dis.

[117] Weidenmaier C., Peschel A., Kempf V.A., Lucindo N., Yeaman M.R., Bayer A.S. (2005). DltABCDand MprF-mediated cell envelope modifications of *Staphylococcus aureus* confer resistance to platelet microbicidal proteins and contribute to virulence in a rabbit endocarditis model. Infect. Immun.

[118] Peschel A., Sahl H.G. (2006). The co-evolution of host cationic antimicrobial peptides and microbial resistance. Nat. Rev. Microbiol.

[119] Ouhara K., Komatsuzawa H., Kawai T., Nishi H., Fujiwara T., Fujiue Y., Kuwabara M., Sayama K., Hashimoto K., Sugai M. (2008). Increased resistance to cationic antimicrobial peptide LL-37 in methicillin-resistant strains of *Staphylococcus aureus*. J. Antimicrob. Chemother.

[120] Friedman L., Alder J.D., Silverman J.A. (2006). Genetic changes that correlate with reduced susceptibility to daptomycin in *Staphylococcus aureus*. Antimicrob. Agents Chemother.

[121] Jones T., Yeaman M.R., Sakoulas G., Yang S.J., Proctor R.A., Sahl H.G., Schrenzel J., Xiong Y.Q., Bayer A.S. (2008). Failures in clinical treatment of *Staphylococcus aureus* Infection with daptomycin are associated with alterations in surface charge, membrane phospholipid asymmetry, and drug binding. Antimicrob. Agents Chemother.

